# Perceptions and practices among Zambian sheep and goat traders concerning small ruminant health and disease

**DOI:** 10.1371/journal.pone.0233611

**Published:** 2020-06-22

**Authors:** Sara Lysholm, Jonas Johansson Wensman, Musso Munyeme, Klara Fischer

**Affiliations:** 1 Department of Clinical Sciences, Section of Ruminant Medicine, Swedish University of Agricultural Sciences (SLU), Uppsala, Sweden; 2 Department of Disease Control, University of Zambia, Lusaka, Zambia; 3 Department of Urban and Rural Development, Division of Rural Development, Swedish University of Agricultural Sciences (SLU), Uppsala, Sweden; University of Lincoln, UNITED KINGDOM

## Abstract

Trade in animals and animal products is a key factor in the transmission of infectious diseases. Livestock traders play an important role in this process, yet there is little knowledge of traders’ perceptions of animal disease or their associated actions. The aim of this study was to investigate perceptions and practices of Zambian small ruminant traders with regard to sheep and goat health and disease. It also analysed how existing perceptions and practices might affect risks of disease transmission through trade. A case study was performed at the two largest small livestock markets in Zambia: the Lusaka market in the capital and the Kasumbalesa market near the border with the Democratic Republic of Congo. Semi-structured interviews with 47 traders performed in April-May and September 2018 represent the core material. Zambian small ruminant traders frequently trade animals that have clinical signs of disease, either because they appear unaware or indifferent to the associated risks, experience financial constraints or assign responsibility for disease prevention to other value chain actors. In their decision about whether or not to sell a visibly sick small ruminant, traders appear to consider whether the clinical sign is perceived as ‘natural’ or the result of an illness, whether the buyer is aware of the animal’s health condition, and whether the animal is sold for consumption or breeding purposes. Traders appear to regard the veterinary certificate required to transport small ruminants in Zambia as proof of health, placing the responsibility for potential disease in traded animals on the veterinary authorities. In their description of a model trader, taking good care of and being sensitive to customer needs was emphasized, indicating that an efficient way to encourage traders to change their behaviour is to influence customer demands. In contrast to the focus in previous studies on identifying and filling knowledge gaps, the present study show that lack of knowledge is not central to why traders engage in disease-transmitting behaviour. Greater awareness of other reasons for certain perceptions and practices could lead to the formulation of risk communication strategies and mitigation measures that are relevant for the local context, as well as alternative strategies for changing trader behaviour.

## Introduction

Market participation and trade among smallholder livestock farmers has received increased recognition in recent years as it is expected to offer an important route out of poverty [1, 2]. However, with growing livestock trade and market engagement, the risk of disseminating infectious diseases also increases. Livestock markets serve as congregation points for animals from various regions, where pathogens can be disseminated, e.g. highly pathogenic avian influenza (HPAI) in multiple locations [3], sleeping sickness (African trypanosomiasis) in Uganda [4] and *peste des petits ruminants* across Africa [5–8]. A significant proportion of the animals sold at urban markets are slaughtered for human consumption, which in the case of substandard or absent veterinary inspections and unhygienic slaughter procedures constitute severe public health risks [9]. Outbreaks of human disease have also been linked to visiting animal markets, e.g. Q-fever [10], SARS [11] and more recently, COVID-19 in Wuhan, China [12].

While much informal livestock trade, especially in rural areas, occurs directly between farmers and customers, it also frequently involves middlemen (traders) who purchase animals from farmers and resell to final customers. Owing to their key position between the producer and the customer, traders can play a central role in the spread or containment of infectious pathogens. Therefore, it is highly relevant for efficient disease control to understand how individuals in this group reason and act in relation to animal health and disease. In rural Zambia, small ruminant trade mostly occurs directly between farmers and customers, but a considerable amount also occurs through traders selling at informal urban markets [9, 13]. The focus of this study was on traders selling animals at these urban markets. Animals that are traded there are often transported over long distances and across several contact points, where sheep and goats from different areas can mix and pathogens be exchanged. Additionally, due to the informal nature of this market chain, market biosecurity and public health protective measures tend to be less controlled by Zambian authorities than in more formalised systems [9]. The health status of small ruminants in Zambia is generally considered good, while several sheep and goat infections with high morbidity and mortality rates are known to be present in neighbouring countries. These include contagious caprine pleuropneumonia (CCPP) [14–17], sheep and goat pox (SGP) [18], and *peste des petits ruminants* (PPR) [7, 14, 16–20]. There is a non-negligible risk of these pathogens being introduced in Zambia, e.g. through informal international trade.

### Previous research on the perceptions and practices of farmers with regard to animal disease

The importance of investigating farmers’ perceptions and practices concerning animal health and disease in order to find effective ways of containing infections, has recently received increased recognition. It is commonly stated within the field of veterinary medicine that farmers fail to comply with recommendations on e.g. disease control owing to a lack of knowledge. Therefore, the focus is frequently on educating farmers about animal diseases [21]. While improving knowledge is important, studies on farmers in different parts of the world indicate that financial and other structural constraints [22], as well as individual farmers’ normative frames of reference [23], sense of responsibility for preventing disease [24], attitudes regarding disease risks [24], and perceived effectiveness of control measures [23, 24] have a greater influence on how farmers act on the basis of disease, treatment and veterinary advice than their factual knowledge.

In recent research, the importance of understanding the concept of “model peers” has been recognised. For example, ideals of what a “good” farmer is have been shown to have a substantial effect on farmers’ perceptions and practices. It is acknowledged that since farmers, like people in general, gain social standing by abiding by norms embedded in their community culture [25–27], it is relevant to study this aspect in order to understand why farmers reason and act the way they do. Studies on livestock farmers in Europe and the US have shown that while there is an emphasis on running a financially successful farm [26] and e.g. low mortality rates [24], it is often judged as equally important to have close emotional ties with one’s animals and be skilled at judging animal health by eye [22, 28, 29]. “Good farmer” ideals are often locally specific, as geographic closeness, regional conditions and local farming practices have been shown to influence the attributes considered desirable [25].

### Previous research on trader perceptions and practices related to animal disease

Results from studies on farmers’ perceptions and practices on animal health and disease should not be uncritically extrapolated to traders, since they generally work in different contexts and have different relationships with the animals. For example, traders typically keep each animal for a limited time and so are unlikely to learn about the specific traits of each animal, which has been highlighted by farmers as an important means of identifying and interpreting animal sickness and health [22, 28, 29]. Furthermore, keeping animals during a limited time is likely to reduce incentives to ensure good long-term animal health. To date, only a limited number of studies have been performed with the purpose of understanding the knowledge, attitudes and practices of traders related to animal health and disease. The majority of these are in the form of quantitative questionnaire studies and cover poultry trade and avian influenza (AI) [30–32] or pig trade and African or classical swine fever (ASF and CSF) [33, 34]. Many of these studies focus on determining factual knowledge levels. A common conclusion drawn is that knowledge among traders is low on various disease features, e.g. clinical signs and transmission routes. Therefore, a collective recommendation is often to take action to improve traders’ knowledge.

A group of studies stand out for adopting a qualitative approach to the study of traders’ perceptions and practices with regard to ASF [35–38]. These studies indicate that traders generally are knowledgeable about major clinical signs and transmission routes [35, 36] and are often aware of their potential role in disseminating ASF. In spite of this awareness, traders frequently engage in activities that can contribute to dissemination of the virus [35–37]. These studies therefore indicate that the risky behaviours employed by traders are unlikely to be due solely to a lack of factual knowledge.

In summary, few studies to date have investigated the relationship between traders’ perceptions and practices on animal health and disease and effects on disease transmission. In light of this research gap, the aim of the present study was to investigate perceptions and practices of Zambian small ruminant traders in relation to sheep and goat health and disease, and to analyse how these might affect risks of disease transmission through trade. To meet these aims, the following research questions guided the work:

1. how do Zambian sheep and goat traders define health and disease in their animals;
2. what factors in the wider trading situation are important for determining traders’ actions with regard to animal disease;
3. what actions do different disease signs warrant from the trader.

## Materials and methods

### Description of the case study

The study was designed as a case study, i.e. a study of “a unit of human activity embedded in the real world; which can only be understood in its context” [39]. The case study consists of the two largest small livestock markets in Zambia: the Lusaka small livestock market, situated in the outskirts of Lusaka in Chibolya township, and the Kasumbalesa small livestock market in the Copperbelt province, adjacent to the border point with the Democratic Republic of Congo (DRC). At the time of the visits, both markets were run by the Small Livestock Association of Zambia (SLAZ), a non-governmental association whose primary purpose is to establish a more organised market system for small livestock in Zambia. The Lusaka small livestock market predominantly trades in goats, pigs, chickens, other fowl and sheep. The market place contains animal pens, a veterinary shop and two slaughterhouses–one for small ruminants and one for pigs. At Kasumbalesa small livestock market, goats, pigs, sheep, chicken and other fowl are sold. There was no veterinary shop or designated slaughterhouse for small ruminants at the time of our visits. Many of the respondents conducted trade at both the Kasumbalesa and Lusaka markets, warranting the treatment of trade at these two markets as one case. In both market places, goats were significantly more common than sheep. According to the respondents, trade is highly seasonal, with more market activity around celebrations and festivities, as well as prior to the due date of school fees.

### Data collection

Data were collected by the first author at the Lusaka small livestock market in April, May and September 2018, and one field visit to Kasumbalesa in September 2018. Authors 2–4 also visited the Lusaka small livestock market in April 2018 and November 2019, allowing the authors to discuss the material together as a group. In all, 21 days were spent at the Lusaka market and four days at the Kasumbalesa market. All data collection occurred in seasons of low trade activity. The main method of data collection was semi-structured interviews with traders present at the market places; 35 traders in Lusaka and 12 traders in Kasumbalesa. A topic guide developed from the research questions was utilised to navigate the interviews with each respondent, while at the same time keeping an openness to new and unexpected information given by the interviewee [39].

During each visit to Lusaka small livestock market, the majority of traders that had not already participated were approached to obtain oral consent for an interview. Around 10% declined to participate, often due to lack of time. Visits at Lusaka market were repeated until saturation in the information coming out from interviews with traders was reached, meaning that no new variations to answers emerged. At Kasumbalesa market, only four visits were made due to time constraints. As the majority of the traders at both markets were men, less than 15% of the respondents in this study are women. All interviews were performed with an interpreter and responses were noted down by hand by the first author. In Lusaka, most of the interviews were performed in the local language Nyanja, while in Kasumbalesa, Bemba was most commonly spoken. In a small number of interviews, the interpreter and the respondent did not speak the same language, and on these occasions other people at the markets interpreted. These interviews were judged to be of lower quality because they were performed with an untrained interpreter, which later was taken into account in the analysis.

To enrich our understanding of the market and trade situation, and to triangulate the responses from traders, interviews with other value chain members were also performed. In Lusaka, five slaughterhouse workers, four transporters, four veterinary shop workers, three SLAZ employees and 20 market customers (of whom two came from the DRC, two were farmers buying for breeding purposes, and 16 were buying for consumption) were interviewed about their reason for visiting the market, their role in the value chain and their perceptions on animal health and disease. At the Kasumbalesa small livestock market, nine market customers, of whom eight were from the DRC and one a farmer from a nearby town, were interviewed. Only individuals aged 18 and above were allowed to participate. To triangulate the information given during interviews [40], and to get a richer understanding of the trading context, observations were made of all activities at the markets. These observations were noted down by hand by the first author in detailed field notes [41].

### Data analysis

Analysis was facilitated by the use of NVivo 12.2.0 software (QSR International, Warrington, UK) and performed by the first author guided by the fourth author. All interviews were thematically coded [42], a process that was guided by the research questions, while being open to new emerging themes. Coding was an iterative process where more detailed themes could be found in the initial broad themes of the research questions, through repeated readings of the material [42, 43] and by theoretically informing later rounds of analysis [43]. For example, early thematic analysis was further refined in the light of the literature on being a ‘good farmer’. This led us to identify a sub-theme in the trader-responses, regarding how traders valued being a good trader. Interviews from other stakeholders and observations of market activities were used to enrich and to triangulate the understanding of emerging themes in the trader-interviews. The quotations in the text are based on written notes from interviews and are thus not verbatim, however the meaning and essence of the respondents’ words, as supplied by the interpreter, have not been altered.

### Ethical considerations

Before starting the work, author 1 and 2 visited SLAZ representatives at the markets to explain the purpose of the study and obtain permission to conduct the research. Prior to commencing the interviews, oral informed consent was obtained from every participant. Care was taken to ensure respondent anonymity and confidentiality by only collecting personal data relevant for the study and never disclosing information related to individual informants to members outside the research team. The study sought and received ethical approval by the ILRI Institutional Research Ethics Committee (ILRI-IREC2018-04).

## Results

The traders in this study came from both rural and urban areas. Traders from rural areas are commonly farmers in addition to their trading activities and will often trade both their own animals and those purchased from other farmers. The area that different rural traders cover in search of sheep and goats for sale varies, but can extend to up to a few hours on foot, by bike or motorized vehicle. Previously bought small ruminants often accompany the traders from one village to the next and are allowed to intermingle with the animals there. Several traders also keep the sheep and goats bought for trade with their own animals at home for some time. Due to high transportation costs, rural traders generally co-organise transport to the markets, resulting in more intermingling.

Prior to transporting animals to markets, a stock movement permit has to be obtained. In brief, this process includes obtaining a certificate from the village headman and the police, who mainly certify that the animals have not been stolen. Furthermore, a certificate from the district council is required, as well as a veterinary clearance to certify that the animals are healthy [44, 45]. The permit, and ideally also the health of the transported animals, is then controlled at police and veterinary checkpoints along the larger roads [38]. Upon arriving at the Lusaka market, the rural traders generally sell their animals to urban traders, who then sell to market customers. More rarely, rural traders sell animals directly to markets customers.

The majority of the sheep and goats sold at the Lusaka and Kasumbalesa small livestock markets are slaughtered for human consumption. In Lusaka, many are slaughtered at the local slaughterhouse, while others are taken to nearby slaughterhouses or slaughtered by the buyers themselves. At the Lusaka slaughterhouse, no ante- or post-mortem inspection was performed at the time of the study visits. Customers who frequently purchase small ruminants for consumption include suppliers for restaurants, bars, hotels, market meat sellers, butchers and people buying for home consumption. A small fraction of purchased animals is kept alive and transported back to farms, mainly for breeding purposes. At both markets, but particularly in Kasumbalesa, traders from neighbouring countries (mainly DRC), purchase small ruminants and transport them back to their home countries where they typically are slaughtered for consumption.

### Traders’ perceptions of small ruminant health and disease

When the respondents were asked what they looked for when purchasing sheep and goats, nearly all of them answered that they primarily look at the size of the animal and whether or not it appears healthy. Many defined being healthy as the absence of various clinical signs, e.g. dull coat, weakness, nasal discharge and diarrhoea. However, the majority mentioned fatness as the most important indication of health, and some respondents even believed that it is impossible for a fat sheep or goat to be ill.

“*I only buy fat and healthy goats*, *they don’t get sick*.*”*

Trader at the Lusaka small livestock market

Several respondents stated that it is rare to witness signs of disease in small ruminants at the market. However, when asked specifically about whether they had witnessed specific clinical signs, e.g. diarrhoea or coughing, several traders admitted that they observed such signs on a regular basis. The clinical signs that were described included diarrhoea, coughing, ocular and nasal discharge, abortion, sudden death and “skin disease”, i.e. an ectoparasitic skin infection manifested by severe pruritus and thickened abraded skin. When asked which specific diseases the respondents had experienced in traded sheep and goats, they generally described clinical signs rather than specific diseases. The only exception was foot and mouth disease (FMD), which was mentioned by some respondents. When asked about causes of the clinical signs, no respondent spontaneously mentioned the possibility of an infectious pathogen. Only when asked specifically whether certain clinical signs could be caused by viruses, bacteria or parasites did a few respondents answer that this was a possibility. Instead, the respondents generally believed that abortion, for example, is invariably caused by beatings or being squeezed together in transport, diarrhoea by feed changes or consuming unsuitable objects, coughing and ocular and nasal discharge by weather changes and dirty environments, skin disease by rain and insufficient cleaning of the pen, and sudden death by stress during transport.

“*With my own goats at home I have problems with skin disease*, *which is caused by rain*. *I treat it by building a shelter for them*. *I don’t understand why they get it*! *It can’t just be because of the rain; rainwater in itself is not harmful*. *There must be something else as well*, *in addition to the rain*.*”*

Trader at the Lusaka small livestock market

At the same time, most respondents were aware that diarrhoea, coughing and skin disease, for example, can be transmitted between animals. Commonly mentioned transmission pathways included airborne spread, eating or drinking together, and coming into contact with animals from far away, e.g. through trade.

“*Trade can contribute to the spread of diseases*. *For example*, *if you buy a goat with diarrhoea and take it home*, *and then the diarrhoea ends up on the grass which another goat eats*, *it can also get diarrhoea*.*”*

Trader at the Lusaka small livestock market

### Trade in small ruminants showing signs of clinical disease

Several respondents stated that they would not engage in trading sick small ruminants. Some attributed this to their own experience and ability to detect diseased sheep and goats, which prevented them from buying and hence also selling sick animals. Other respondents had a humbler view of their ability to identify disease in small ruminants, but believed that they did not keep the animals long enough for them to develop disease. However, the most commonly cited reason for animals not being sick in the care of the traders was the requirement to obtain veterinary clearance as part of the stock movement permit prior to transporting the animals to markets. Many regarded this process as a guarantee of health and the absence of disease in all animals that were being traded at the markets.

“*It would be possible for trade to contribute to spreading animal diseases*, *but not with the stock movement permit system*. *The permit ensures that only healthy animals are traded*.*”*

Trader at the Lusaka small livestock market

Some respondents argued that the system with stock movement permits means that excluding sick animals from trade is the responsibility of the veterinary authorities, not the members of the value chain.

“*It is the responsibility of the council and the veterinarians to prevent diseases from getting to Chibolya*.*”*

Trader at the Lusaka small livestock market

However, the interviews indicated that the process for acquiring a veterinary clearance varied considerably between districts. According to a small number of informants, the veterinarian in their district just signs the certificate without seeing the animals. Others stated that the veterinarian performs a visual examination of all the animals as well as a physical examination of a selected few. The most common answer was that the veterinarian performs a visual examination of the whole group (not examining individuals) and then issues the certificate. In addition, at veterinary checkpoints during transportation, several traders reported that while the permit itself is controlled, the animals themselves are only looked at occasionally. Also, according to several informants, as corruption is widespread in Zambia, bribing personnel at checkpoints is common, which further impedes the efficiency of the stock movement permit system.

Several interviewees initially stated that they never had and never would engage in trade with animals that were sick. Many stressed that it could be harmful for their business as they risk losing customers if it was detected and exposed, or that they could get in trouble with the Zambian authorities. Others described the sale of sick animals as immoral and that it conflicted with their religious beliefs, while others refrained because it “felt wrong” or because of a reluctance to contribute to disseminating disease among sheep and goats or to consumers.

“*I would never sell a sick goat*, *no matter how mild the disease was*. *If I did*, *I would contribute to spreading the disease or putting people at risk of getting sick*.*”*

Trader at the Lusaka small livestock market

However, with several respondents, a different picture emerged when enquiries were made about whether it is acceptable to sell sheep and goats with specific clinical signs. The majority of the informants believed that selling sheep and goats that are coughing and have nasal and ocular discharge is acceptable, since they either perceived these clinical signs to be very mild or to not stem from disease, but rather from a dirty environment or the weather. According to some, trading sheep and goats with coughs or ocular and nasal discharge is acceptable, but only when it is not caused by disease. When asked to explain how they could be sure whether a clinical sign was due to a disease or not, some admitted that they could not be sure, while others stated that they examined the general health appearance of the small ruminant in question. More specifically, if a coughing animal otherwise appears healthy (i.e. it is not thin or weak for example), they would deduce that it is fit for trade. However, reasoning varied significantly concerning the trade of sheep and goats with diarrhoea and what traders referred to as skin disease. Some believed that it is acceptable since they perceived these clinical signs to be mild and easily treatable, and in the case of diarrhoea, that it is not caused by disease but rather by ingesting unfamiliar feeds or unsuitable objects. Others were of the opinion that selling sheep and goats with diarrhoea or an ectoparasitic skin infection is deeply immoral because they are severe, sometimes deadly diseases and are often difficult to treat.

Upon probing, it turned out that several interviewees were prepared to sell sick sheep and goats with any clinical signs of illness. Some participants considered selling sick animals to be an acceptable practice, provided the buyer is made aware of the health status of the small ruminant. Additionally, a few respondents were comfortable with trading diseased animals provided they are sold for consumption and not for keeping by farmers, as they believed that the disease cannot be transmitted to humans, only to other sheep and goats. Two traders stated that since customers at the markets did not care about whether the animals they purchased were sick or not, they were also choosing to overlook it.

“*Kasumbalesa is the end point for the goats and the sheep*. *The majority are being sold for consumption and then it does not matter*, *nobody cares whether they are sick or not*.*”*

Trader at the Kasumbalesa small livestock market

However, several respondents expressed feelings of moral conflict, but did not see any other financially feasible alternative than selling the animal. The possible negative financial impacts mentioned by the traders included lost income for the diseased small ruminant as well as costs associated with transporting the sick animal away from the market.

“*I know selling sick animals is wrong*, *but what can I do*? *I don’t have the ability to treat them like I would if my goats at home got sick*. *I can’t transport them back home because it’s too expensive*. *My only option is to sell them to avoid losing money*.*”*

Trader at the Kasumbalesa small livestock market

A few respondents stated that they would attempt to sell the animal quickly after discovering signs of disease to avoid it succumbing to illness prior to being sold. One trader disclosed that he would slaughter the sick animal and sell the meat to avoid lost income, since he would get paid more for the meat than for an animal displaying clinical signs of illness.

The majority of respondent traders reported avoiding purchasing sick sheep or goats. This was mainly out of fear of financial losses, either if the sick animal died or if it transmitted the disease to the other sheep and goats. Nonetheless, some interviewees mentioned that they sometimes buy small ruminants with diseases that they perceive to be mild and/or treatable at a low cost, such as ocular and nasal discharge or skin disease. They then treat the illness and make a profit by selling the animal at a usual price.

When small ruminants with signs of disease appear at the market, perceptions regarding the correct mode of action varied significantly. None of the traders reported the disease to market officials or authorities. A few informants stated that they would keep the sick animal for various lengths of time to allow it to recover. Some mentioned buying medicines, but this was uncommon. The most common action mentioned was to proceed with the sale of the sick animal without attempting treatment or other further actions. During the study visit to the Lusaka small livestock market, no preventative measures were instituted for the market as a whole, but according to SLAZ personnel, there were plans to introduce such measures, primarily in the form of training workshops for value chain members. When individual traders were asked whether they took disease prevention measures at the market, the majority replied that they did not, although some mentioned providing feed and water of good quality for their animals. In spite of this, all the informants took at least one disease prevention measure, most commonly cleaning the pen. However, this was not necessarily regarded as a preventive measure, but was used to attract customers.

### Traders’ perceptions of public health-related hazards and the practices that cause them

Several interviewees were aware of the hazards associated with the consumption of sick animals, primarily the risk of falling ill from the same disease, but many also feared residues from previous medical treatments. However, several respondents would consume meat from sick animals, despite being aware or at least suspicious of the risks associated with this.

“*Every cold season my village experiences a strange outbreak that affects all grazing animals*. *They suddenly collapse and die*, *blood comes out of their noses and their bodies become bloated*. *When we open the carcasses*, *their lungs are filled with blood*. *We haven’t asked the veterinarians for help because they are located very far away from us*. *We usually eat the carcasses to avoid losses*.*”*

Trader at the Lusaka small livestock market, describing the consumption of possible anthrax cases

Some participants explained their readiness to consume products from sick animals by not perceiving various clinical signs as the result of disease, but rather due to what is perceived as natural processes. Others expressed confidence in their own ability to determine whether the clinical sign poses a health risk to the consumer or not. A few respondents described a strong love for eating meat and an unwillingness to waste food.

“*Have we ever slaughtered an animal because it was sick*? *Yes*, *many times*, *we ate the meat and nothing happened*! *It’s because we love meat so much*. *We’d rather take the risk of getting sick than waste food*.*”*

Trader at the Lusaka small livestock market

Furthermore, several participants believed that they have taken protective measures that remove the risk of food-borne infections, for example by always boiling meat before consumption or discarding certain organs (e.g. the intestines if the animal has diarrhoea, lungs if it is coughing etc).

A practice that perhaps poses greater risks to public health than slaughtering and consuming diseased sheep and goats is consuming animals that have died on their own. Most respondents stated that although they knew several people who ate animals that had died on their own, they would never do this themselves, either due to a strong feeling of repulsion, religious beliefs or out of fear of succumbing to the same illness as the animal. Respondents who admitted to consuming self-dead animals often justified this by their great love of meat and unwillingness to waste food. Additionally, several misconceptions were identified among the respondents, e.g. that boiling the meat ensures that disease cannot be spread to the consumer.

“*Eating the meat from a sick animal or from an animal that has died on its own is okay as long as the meat is boiled first*.*”*

Trader at the Lusaka small livestock market

At the Lusaka and Kasumbalesa markets, there were designated locations for disposing of dead animals, and thus the sale of self-dead animals was not an accepted practice. Most of the traders interviewed stated that they had never and would never sell the body of an animal that had died on its own, either because it is immoral or forbidden according to their religion. However, a few traders admitted to selling bodies of animals that had died on their own, and several respondents stated that some buyers visit the markets solely to buy dead animals at cheap prices. One transporter described how he would sell animals that had died during transport, most commonly to meat vendors outside bars that specialised in selling meat to people on their way home after a night out.

“*I sometimes sell the bodies of animals that die during transport for a cheap price*. *I do it because I feel bad for the owner who needs the income from the sale*. *Usually they are sold to owners of barbeque stands outside bars*.*”*

Transporter and trader at the Lusaka small livestock market

### Being a “good trader”

When the respondents were asked about being a “good trader”, it was clear that being perceived as good by their peers was considered important. The most commonly mentioned characteristic of a good trader was being good at dealing with people, more specifically at establishing favourable relationships with customers, even if they are rude, and being a skilled price negotiator. Many also emphasised the importance of keeping the trade environment clean because this attracts customers.

“*A model trader is someone who can relate to customers and be good to them*, *even if they are rude back*. *A good trader will also keep the environment clean to attract customers”*

Trader at the Lusaka small livestock market

Some also defined a “good trader” as someone who keeps animals that look healthy. However, this was mainly emphasised because it attracts market customers rather than because it is beneficial from an animal health perspective.

“*A good trader is someone who trades with animals that are clean and look fat and healthy*, *since this is what attracts customers”*

Trader at the Lusaka small livestock market

## Discussion

The Lusaka and Kasumbalesa small livestock markets congregate small ruminants from different regions across Zambia and therefore pose major risks for disease dissemination. Furthermore, as the majority of animals sold enter the human food chain, many of which are slaughtered without ante- or post-mortem inspections, the Zambian small ruminant trade system poses a significant public health risk. The results of this study show that the risks of disease dissemination and to public health are increased by traders’ practices. The majority of the respondent Zambian sheep and goat traders were found to be either engaged in trade with diseased small ruminants or believed that it is more or less acceptable to do so, at least under certain circumstances. Several traders restricted themselves to only engaging in trade with sick sheep and goats when the clinical sign is considered mild or due to natural processes, or to only selling diseased small ruminants for consumption and not to farmers. Many interviewees reasoned that the Zambian stock movement permit system absolves them from responsibility for preventing disease dissemination, since the animals have been certified as healthy by a veterinarian and are subsequently checked at veterinary checkpoints enroute to the markets. However, this study indicates that the stock movement permit system is not adequately implemented to detect disease, and previous research has demonstrated that the veterinary checkpoints are often undermanned and commonly bypassed [38]. It appears as though the transfer permit system has primarily made value chain members focus on obtaining the correct paperwork, with limited reflection on why this is important, rather than actively taking steps to prevent the dissemination of disease.

The respondents’ motivation for participating in trade with sick small ruminants can be grouped into three different categories. The first category expressed feelings of moral conflict, but lacked the financial means to withhold the animals from sale or transport them away from the market. The second category were either unaware of the associated hazards or appeared unwilling to accept them as such, e.g. by (strategically or otherwise) downplaying the relevance of certain clinical signs or avoiding to classify them as signs of disease. The third category attempted to assign the responsibility elsewhere, either to buyers by making them aware of the animal’s health condition or to the authorities who are responsible for issuing the pre-transportation veterinary certificate. In previous research among traders, lack of knowledge is a commonly stated reason for risky behaviours such as trading sick animals [30–32, 34]. The traders in the current study trade diseased small ruminants for a span of different reasons and would therefore be likely to need different forms of intervention strategies to stop this risky behaviour. The results of this study indicate that only some traders would be significantly affected by a knowledge improvement campaign. Consequently, there is a clear need for research and outreach projects to adapt their agendas and move beyond simply attempting to improve knowledge among value chain actors.

While research on traders’ perceptions and practices related to animal health and disease is limited, a significant number of studies have been performed with the same focus on livestock farmers. Similar to studies focusing on farmers’ perceptions of animal welfare and disease, the present study indicates that the wider culture of trading and being a respectable trader in the eyes of one’s peers is an important driver for how traders reasoned and behaved in relation to sick animals. However, this study clearly shows that the traders relate to a different culture and context when creating their perceptions on animal health and disease than the farmers in studies on the cultural shaping of farmer behaviour. While farmers often highlight the importance of establishing a relationship with their animals [22, 28–29], the traders in the present study emphasised their ability to build relationships with people, i.e. being good at dealing with clients. Hence, being a good trader has less to do with how animals are cared for and more to do with maintaining good customer relations. Influencing client demands is therefore likely to be an efficient way of adjusting traders’ perceptions and practices.

The study visits to both markets were conducted at times of comparatively low trade activity. Visiting the markets during the peak trade season would have increased the possibility to observe trader behavior during disease outbreaks, as more animals and people present means increased risk of disease dissemination. At the time of the study visits, both the Lusaka and Kasumbalesa markets were run by the Small Livestock Association of Zambia (SLAZ). Several of the respondent traders, slaughterhouse workers, transporters etc. were members of the association. While this may have reduced respondents’ willingness to supply information that could project a negative image of the organisation, we do not believe that this had any significant bearing on their ability to answer the research questions. In this study, the respondent traders were treated as a homogenous group, and differences related to gender, country of origin etc. were not investigated. These are relevant aspects for exploration in future larger studies.

## Conclusions

In conclusion, while lack of knowledge is a common explanation in the existing scientific literature as to why traders, through their actions, engage in behaviours that can lead to disease contamination and spread, this study indicates that the reality is far more complex than that. This warrants a shift in the focus of research and outreach projects from simply identifying knowledge gaps to understanding the underlying reasons and drivers behind certain behaviours. Through increased awareness of traders’ perceptions and practices, risk mitigation measures and communication strategies relevant for the local context can be formulated. This could reduce the risks of trade and market-associated disease dissemination, which would lead to improved small ruminant health and smallholder farmer livelihoods in Zambia.

## Supporting information

S1 File An overview interview guide for the topics that were covered in the semi-structured interviews with traders. (DOCX)
