# Supplementary material for: Perceptions and practices among Zambian sheep and goat traders concerning small ruminant health and disease
Source: PLoS One. 2020 Jun 22;15(6):e0233611. doi: 10.1371/journal.pone.0233611 (PMC7307758; doi:10.1371/journal.pone.0233611)
Supplement: S1 File — (DOCX) [file pone.0233611.s001.docx]

| **Department of Clinical Sciences** |  |
| --- | --- |

**Interview topic guide - Traders**

1. **Respondent and market information**

Gender of the respondent

Market name and location

Short description of the interview situation
*Does the respondent seem comfortable/uncomfortable/stressed etc? Is the respondent interviewed in private or with an audience? Etc.*

1. **General**

What is your first language?

What/which languages do you speak?

Where do you live?

Are you a farmer? If YES; what are you a farmer of?

How often approximately do you come here to sell sheep and goats?

Do you trade anything else other than sheep and goats?

Do you visit any other markets than Chibolya/Kasumbalesa?

1. **Trade routines**

Do you trade with sheep and goats that you have reared yourself?

Do you trade with sheep and goats that you have bought from other farmers?

ASK ONLY if the respondent is trading with sheep and goats that he or she has bought from other people :

- From what kind of people are the sheep and goats that you trade? Farmers? Traders? Others?
  *Probe for whether this is how it is usually done and approximate proportion of the different alternatives, if the respondent mentions several*
- From where do you buy/barter sheep and goats to trade with?
  *District, closest town, if possible village
  Listing and ranking from most to least common*
- Where are the sheep and goats that you are trading today from?
  *Note district, closest town and if possible village*
- Do you take the sheep and goats with you when you move between the villages?
- How do you transport the sheep and goats between villages?
- When you come to a new village, do you let the sheep and goats intermingle with sheep and goats there?

What happens to the sheep and goats after they have been sold?
*Slaughter, breeding etc
Listing + ranking*

What happens to the sheep and goats that have not been sold?

1. **International trade**

Do you know or have you heard of other traders who gets (buys/barters) sheep and goats from other countries and takes them to Zambia?
If YES;

- Can you describe how they usually do this?
  *Probe for whether they are quarantined etc.*
- How common would you say that this is?

Do you know or have you heard of other traders who sell to other countries?
If YES;

- Can you describe how they usually do this?
  *Probe for whether they are quarantined etc.*
- How common would you say that this is?

Are all sheep and goats that you are selling here today from Zambia?

If NO;

- Specify which country they are from

Have you ever (bought/bartered) sheep or goats from another country?
If YES;

- From which country?
- Can you describe the process
  (*probe for quarantine etc*)
- How often approximately has this happened?

Have you ever sold to another country?

If YES;

- To which country/countries?
- Can you describe the process
  (*probe for quarantine etc*)
- How often approximately has this happened?

1. **Animal health**

How can you know if a goat or a sheep is sick?

What signs of disease do you see in goats or sheep that you are trading?

*Listing, ranking from most to least common*

What do you do if you see signs of sickness in goats or sheep that you are trading?

If you noticed that a goat or sheep had runny eyes and nose, what would happen?

What causes runny eyes and nose in goats or sheep?

If you noticed that a goat or sheep was coughing, what would happen?

What causes coughing in goats or sheep?

If you noticed that a goat or sheep had difficulty breathing, what would happen?

What causes difficulty breathing in goats or sheep?

If you noticed that a goat or sheep had diarrhea, what would happen?

What causes diarrhea in goats or sheep?

If you noticed that a goat or sheep had aborted, what would happen?
*Probe for what happened with the foetus and the foetal membranes*

What causes abortion in goats or sheep?

Has it ever happened to you that one or several of the sheep and goats that you were trading died suddenly?
If YES;

- What happened then?
- How did you dispose of the body?

What causes sudden death in goats or sheep?

Which diseases would you say are the most common in goats and/or sheep at the market?

*Listing, ranking*

1. **Trade of sick sheep and goats**

Has someone ever sold or given you a sick sheep or goat?

Do you know of traders who buys or who have bought a sick sheep or goat?

Do you know or have you heard of other traders that have sold/traded/bartered sheep or goats that were sick?

If YES,

- How common would you say that this is?

Have you ever sold a sheep or goat that you thought was sick?
If NO,

- Why is this?

If YES;

- Approximately how often has this happened
- What signs of disease were these animals showing?

Are there any signs of disease that sheep and goats can have and it is still ok to sell them?

Are there any signs of disease that, if a sheep or a goat has it, it is inappropriate to sell it?

Are there any signs of disease that sheep and goats can have and it is still ok to buy them?

Are there any signs of disease that, if a sheep or a goat has it, it is inappropriate to buy it?

Are there any situations when it is OK to sell a sick sheep or goat?

Do you know, or have you heard of other traders who sell the body of dead sheep and goats?
If YES;

- How common do you think it is?

Have you ever sold the body of a sheep or goat that had died for some reason, for example due to disease?
If NO,

- Why not?

If YES;

- Approximately how many times?

Are there any circumstances where you do not think it is ok to sell the body of a dead sheep or a goat?

Are there any circumstances where you think it is ok to sell the body of a dead sheep or a goat?

Why do people sell sick sheep and goats?

Why do people sell dead sheep and goats?

Why do people buy sick sheep and goats?

Why do people buy dead sheep and goats?

What would encourage you to refrain from selling a sick animal (if the respondent does so)?

What would encourage you to refrain from selling the body of a dead animal (if the respondent does so?)

Who is responsible to prevent trade with sick sheep and goats?

1. **Control measures**

Are you taking any measures to prevent diseases in sheep and goats that you are trading?
If NO;

- Why not?

If YES;

- Which measures?

Do you ever treat sheep and goats that you are trading with medicines?
If NO;

- Why not?

If YES;

- What kinds of medicines?
- Where do you get them from?
- Do you seek advice before treatment and if yes : from whom?
- What diseases or symptoms do you treat with which medicines?

Is there someone that you can seek advice from if you notice signs of disease in your sheep and goats?

*If YES; Probe for profession*

1. **Reporting disease**

Is there a person or an institution that you can report to if you find a sick sheep or goat?

Have you ever reported a sick sheep or goat to [person/institution mentioned above]?
If NO;

- Why not?

If YES;

- Approximately how many times?
- What signs of disease were those animals showing?
- To whom did you report?

Are there situations when you feel it is necessary to report sickness in sheep and goats?
If YES;

- Describe these situations
  *(Probe for clinical signs, many animals sick etc)*

Are there situations when you do not feel it is necessary to report sickness in sheep and goats?
If YES;

- Describe these situations
  *(Probe for clinical signs, many animals sick etc)*

What would encourage you to report sickness in sheep and goats to veterinarians/authorities?

1. **Knowledge**

Can you give some examples of how infectious diseases can be spread?

Can trade contribute to spreading infectious diseases in sheep and goats?

If YES;

- In what ways do you think that trade can contribute to the spread of infectious diseases?

Are there any risks with trading sheep and goats between countries?
If YES;

- What are those risks?
- If the respondent has admitted to trading with other countries, ask if he/she reflects on this when he/she is conducting international trade

Are there any risks with selling sheep and goats that are sick?
If YES;

- What are those risks?
- If the respondent has admitted to selling sick sheep or goats, and says he/she thinks there are risks involved, ask if he/she reflects on this when he/she is selling sick sheep and goats

Are there any risks with selling sheep and goats that have died on their own?
If YES;

- What are those risks?
- If the respondent previously has admitted to selling sheep or goats that has died, and says he/she thinks there are risks involved, ask if he/she reflects on this when/he/she is selling self-dead sheep and goats

Are you familiar with a disease called peste des petits ruminants/?
If NO;

- Give short information

If YES;

- Can you briefly describe the signs of disease that it causes?
- What species are affected?
- Can you describe how it is transmitted
- What would you do if you saw sheep and/or goats that you thought had PPR?

Are you familiar with a disease called PPR?
If NO;

- Give short information

If YES;

- Can you briefly describe the signs of disease that it causes?
- What species are affected?
- Can you describe how it is transmitted
- What would you do if you saw sheep and/or goats that you thought had PPR?

Have you heard of a disease called foot and mouth disease?
If NO;

- Give short information

If YES;

- Can you briefly describe the signs of disease that it causes?
- What species are affected
- Can you describe how it is transmitted?
- What would you do if you saw sheep and/or goats that you thought had FMD?

Have you heard of a disease called FMD?
If NO;

- Give short information

If YES;

- Can you briefly describe the signs of disease that it causes?
- What species are affected
- Can you describe how it is transmitted?
- What would you do if you saw sheep and/or goats that you thought had FMD?

1. **Stock movement permit and other laws or regulations**

Are there any laws or regulations that regulate how sheep and goats are allowed to move within Zambia?
If YES;

- Can you describe them?

Are there any laws or regulations that regulate trade of sheep and goats between Zambia and Tanzania?

If YES;

- Can you describe them?

1. **Zoonotic diseases**

Are there diseases that sheep and goats can transmit to humans?

If YES;

- Can you mention some diseases that both humans and sheep/goats can get sick of?
- How can you be infected by sheep and goat diseases?
- Are there any measures that you can take to protect yourself from getting infected by sheep and goat diseases?

1. **Outbreaks**

Have you ever experienced outbreaks of disease in sheep and goats that you are trading? (Several animals falling ill showing the same symptoms)

If YES;

- How often
- When was the last time?
- Disease signs?
- What species were affected?
- How many individuals were affected?
- Were other households in the village also affected?
- Did people also get sick?
- What did you do?

Have you ever experienced an outbreak of runny eyes and nose, coughing and diarrhea?

If YES;

- How often
- When was the last time?
- Disease signs?
- What species were affected?
- How many individuals were affected?
- Were other households in the village also affected?
- What did you do?

**Thank you! Do you have any questions for me?**
